# Supplementary material for: Single-Cell Sequencing Analysis Identified ASTN2 as a Migration Biomarker in Adult Glioblastoma
Source: Brain Sci. 2022 Oct 30;12(11):1472. doi: 10.3390/brainsci12111472 (PMC9688571; doi:10.3390/brainsci12111472)
Supplement: Supplementary file 1 [file brainsci-12-01472-s001.zip › brainsci-1979941-supplementary.pdf]

A

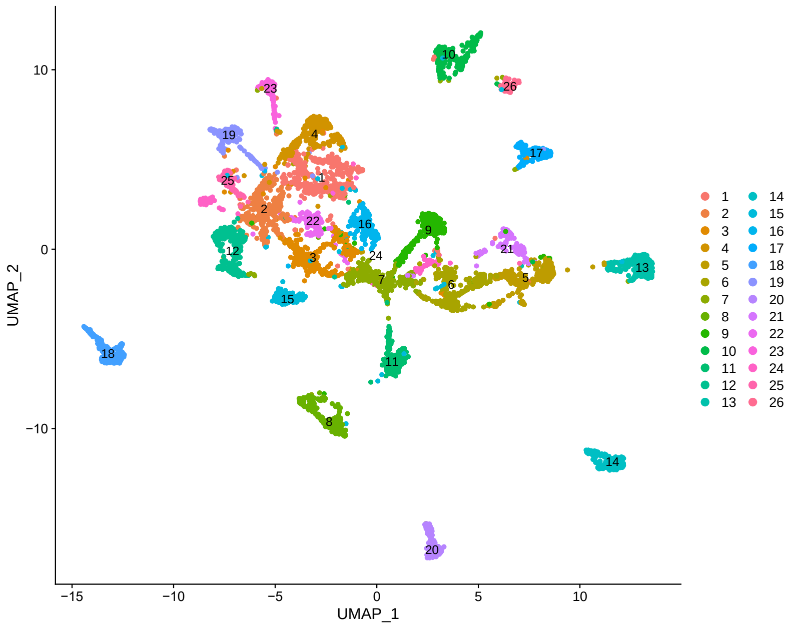

B

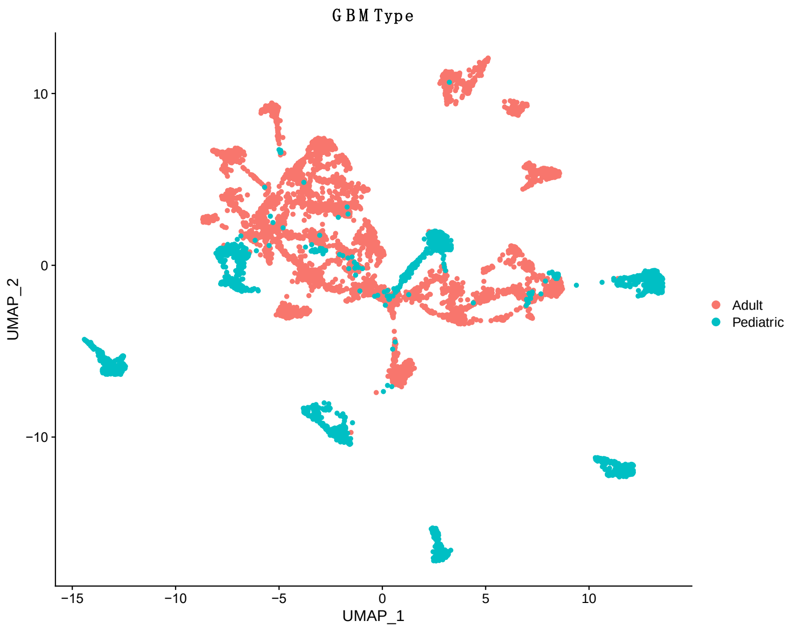

Supplementary figure S1. Integrated analysis of adult and pediatric GBM cells. (A) UMap dimensionality reduction of clustering results. The X-axis and Y-axis represented the two dimensions of the UMap dimensionality reduction space. The points in the figure were cells, and all cells were colored separately according to the cluster labels. (B) Distribution of cell clusters in adult group and child group. The results showed that the adult GBM cells accounted for more cell clusters.

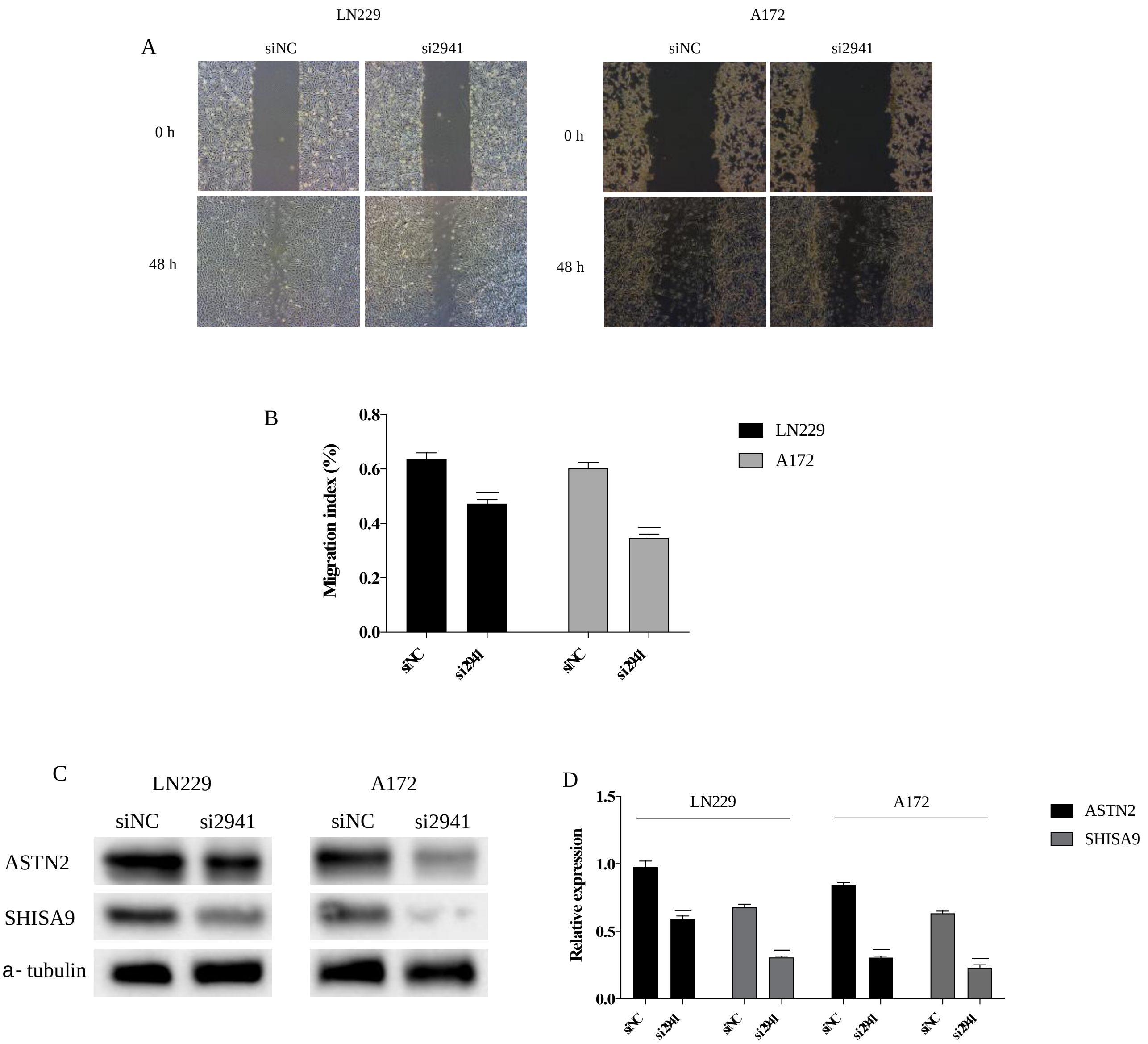

Supplementary figure S2. ASTN2 increases the migration ability of GBM cells. (A) and (B) Wound healing assay showed that the control group cells healed the wound to a greater extent than the ASTN2 interference group cells after 48h. (C) and (D) Western blot showed that the expression of SHISA9 decreased after down-regulation of ASTN2.
